# SpidermiR: An R/Bioconductor Package for Integrative Analysis with miRNA Data

**DOI:** 10.3390/ijms18020274

**Published:** 2017-01-27

**Authors:** Claudia Cava, Antonio Colaprico, Gloria Bertoli, Alex Graudenzi, Tiago C. Silva, Catharina Olsen, Houtan Noushmehr, Gianluca Bontempi, Giancarlo Mauri, Isabella Castiglioni

**Affiliations:** 1 Institute of Molecular Bioimaging and Physiology National Research Council (IBFM-CNR), Segrate (Mi) 20090, Italy; gloria.bertoli@ibfm.cnr.it (G.B.); alex.graudenzi@unimib.it (A.G.); 2 Interuniversity Institute of Bioinformatics in Brussels (IB)2, Brussels 1050, Belgium; antonio.colaprico@ulb.ac.be (A.C.); colsen@ulb.ac.be (C.O.); gbonte@ulb.ac.be (G.B.); 3 Machine Learning Group (MLG), Department d’Informatique, Universite libre de Bruxelles (ULB), Brussels 1050, Belgium; 4 Department of Genetics Ribeirao Preto Medical School, University of Sao Paulo, Ribeirao Preto, Sao Paulo 14049-900, Brazil; tiagochst@gmail.com (T.C.S.); houtana@gmail.com (H.N.); 5 Department of Neurosurgery, Henry Ford Hospital, Detroit, MI 48202, USA; 6 Department of Informatics, Systems and Communication, University of Milan-Bicocca, Milan 20125, Italy; mauri@disco.unimib.it; 7 SYSBIO Centre of Systems Biology (SYSBIO), Milan 20126, Italy

**Keywords:** microRNA, network, protein, gene

## Abstract

Gene Regulatory Networks (GRNs) control many biological systems, but how such network coordination is shaped is still unknown. GRNs can be subdivided into basic connections that describe how the network members interact e.g., co-expression, physical interaction, co-localization, genetic influence, pathways, and shared protein domains. The important regulatory mechanisms of these networks involve miRNAs. We developed an R/Bioconductor package, namely SpidermiR, which offers an easy access to both GRNs and miRNAs to the end user, and integrates this information with differentially expressed genes obtained from The Cancer Genome Atlas. Specifically, SpidermiR allows the users to: (i) query and download GRNs and miRNAs from validated and predicted repositories; (ii) integrate miRNAs with GRNs in order to obtain miRNA–gene–gene and miRNA–protein–protein interactions, and to analyze miRNA GRNs in order to identify miRNA–gene communities; and (iii) graphically visualize the results of the analyses. These analyses can be performed through a single interface and without the need for any downloads. The full data sets are then rapidly integrated and processed locally.

## 1. Introduction

Gene regulatory networks (GRNs) play a crucial role in many key biological processes, such as cell differentiation, metabolism, cell cycle, and signal transduction. For instance, when a pathological process is ongoing, the dynamics of GRNs are most likely altered. Thus, the differences observed in GRNs in healthy and pathological conditions may reveal the mechanisms behind disease onset and progression.

A wide number of computational tools have been developed for analyzing and displaying GRN aided by the ever-increasing public availability of (big) data.

GeneMANIA [1] is one of the most popular web-based tools for studying validated biological networks. It enables different types of protein-protein and gene–gene interactions and networks to be predicted and visualized. Initially released in 2010, it currently indexes more than 2000 association networks, and maps more than 500,000,000 interactions from nine different organisms, extracted from a large number of publicly available sources. 

MicroRNAs (miRNAs), which are small non-coding RNAs, are emerging as key drivers in the regulation of GRNs [2,3]. This is evidenced by the growing number of papers on this topic (from approximately 200 articles published in 2010 to 600 in 2015). MicroRNAs are particularly appealing as non-invasive tools for disease diagnosis, prognosis, and also therapy, since they have been found to be stably expressed in biofluids (such as serum and plasma) in several pathologies [4,5].

Some studies have provided insights into miRNA–gene regulation, showing that miRNAs tend to target highly connected genes in cellular networks, thus identifying miRNA–gene communities [6,7]. Since community structure conveys important information on specific functions [8], several community detection algorithms have been implemented to identify community structural properties, e.g., centrality measures, path length properties, and vertex and edge attributes.

Degree centrality (i.e., in the graphical representation of GRN, the number of neighboring vertices to which a node is directly connected) has been widely used for the analysis of biological networks [9]. It enables high-density nodes to be detected and ranked, e.g., proteins, genes, and miRNAs.

Several online resources provide collections of multiple databases that enable biologists to easily identify the biological functions and regulatory relationships between a group of known/putative miRNAs and validated/predicted target proteins, genes, and also associations with diseases or drugs (see, e.g., miRTAR [10], miRwalk [11], DIANA [12], Miranda [13], PicTar [14], TargetScan [15], miRandola [16], miR2disease [17], and Pharmaco-miR [18]). However, these resources need to be imported into other software tools for processing, tabulation, analysis, and graphing.

To overcome these limitations, more advanced tools have been recently made available to the scientific community such as collections of predicted and validated miRNA–target interactions (e.g., miRNATap [19], multiMiR [20], and MAGIA^2^ [21]). Although each of these tools supports the user with particular features, only MAGIA^2^ is able to automatically integrate the information on miRNA interaction networks with GRNs but with some limitations. For instance, there is no information on validated protein–protein or gene–gene interactions, or miRNA associations with diseases and drugs. MAGIA^2^ only provides information on the interaction between transcription factors and the genes regulated by those transcription factors, omitting any other information regarding gene–gene interaction.

In this work, we describe a new software tool, namely “SpidermiR”, which automatically integrates with GRNs the information on miRNA interaction networks, and the association with drugs and diseases. It enables users to query, download, analyze, and visualize miRNA data with respect to specific GRNs, including different types of gene–gene (protein–protein) interactions (e.g., co-expression, genetic interactions, pathways, physical interactions, and shared protein domains). 

SpidermiR has already been integrated with the most comprehensive repository of human cancer molecular and clinical data, The Cancer Genome Atlas (TCGA) [22,23,24], collecting 33 different tumor types by sampling across hundreds of cases per tumor type. 

Most studies on TCGA data have focused on differentially expressed genes (DEGs) among different groups of tissue samples, and these analyses have not yet been able to fully reveal the relationships among genes or their dependency with miRNAs. Thus, the identification of miRNA-gene communities enriched by DEG, as is possible with SpidermiR, could simplify the problem of data interpretation and clarify the role of miRNAs in the onset and development of a specific cancer.

SpidermiR is available as open source and open development software in the bioconductor platform and thus is easily visible and accessible to an active user community, thereby promoting the reproducibility and the transparency of results.

## 2. Results and Discussion

In this section, we present the key features implemented in the SpidermiR package, and highlight the advantages of this software compared to other packages currently available to the scientific community with similar purposes.

We then present two case studies that will help in clarifying the utility of SpidermiR for users. The two case studies are shown in addition to the executable R code in the online documentation [25,26].

### 2.1. Key Features

**Figure 1:**
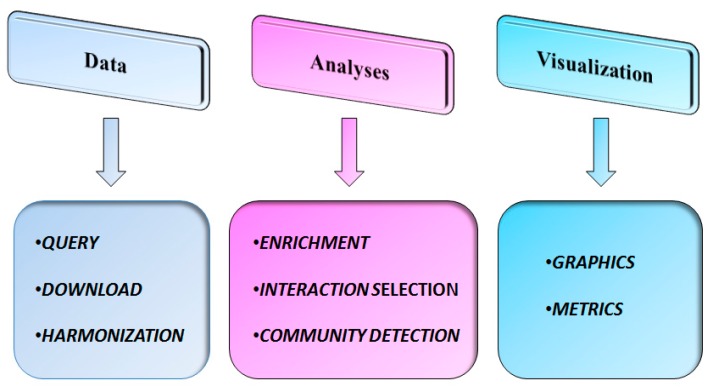
The key features of SpidermiR package.

Figure 1 highlights the key features of SpidermiR.

SpidermiR allows users to: (i) query and download GRNs from GeneMANIA and miRNAs from validated and predicted repositories, and harmonize annotations for miRNAs, genes, and proteins (query/download/annotation harmonization); (ii) integrate miRNA data with GRNs in order to obtain miRNA-regulated networks (miRNA–gene–gene and miRNA–protein–protein interactions) (enrichment), analyze miRNA GRNs to select specific interactions (interaction *s*election) and identify miRNA–gene communities (community detection); and (iii) graphically visualize and quantitatively summarize the miRNA-GRNs (graphics/metrics).

These analyses can be performed without having to navigate and access different web-based databases, without the need to download data, and by integrating and locally processing the full data sets in a short time. 

SpidermiR is implemented as an R package, licensed under the General Public License (GPLv3). It is freely available, through the Bioconductor repository, at [25,26].

### 2.2. Benchmarking

SpidermiR offers easy access to both GRNs and miRNAs, and integrates this information with differentially expressed genes obtained from TCGA. It has several advantages with respect to other tools for integrative network analysis with miRNA data: the main difference is that in the competing tools, the role of miRNAs in the gene network has scarcely been considered and assessed.

As specified in the introduction, GeneMANIA is a web portal enabling users to build and visualize a composite gene–gene (both predicted and validated) or protein–protein (validated) functional interaction network. Currently, there are no functions that allow users to download these data in R or in Bioconductor in order to analyze them and integrate the networks with other information. 

miRNAtap is a software package integrated in Bioconductor, solely focused on four predicted miRNA–gene databases (Miranda, PicTar, TargetScan, miRandola), and it integrates miRNA target prediction from different sources, aggregating them with various methods. Compared to miRNAtap, SpidermiR provides more features such as miRNA pharmaco/disease association, network data, analysis function, and miRNA–gene databases.

MultimiR is an R script that provides users with both predicted data from eight external databases (DIANA, EIMMo, MicroCosm, miRanda, miRDB, PicTar, PITA, and TargetScan), and validated miRNA–gene interactions from miRecords, miRTarBase, and TarBase. It also provides the users with both disease and drug associations with miRNAs from several databases (miR2Disease, Pharmaco-miR and PhenomiR). Although MultimiR has a greater number of predicted databases it does not allow the user to integrate the GRN and TCGA data. Consequently, although several tools have recently been developed to improve miRNA–gene identification almost none of them address the complex problem of reconstructing miRNA–gene target–genes in the different network types. 

In fact, the association miRNA–gene is not sufficient to study miRNA activity. miRNA activity should be studied as part of a compound system where the single elements can interact among them on multiple levels. To understand such a complex system, it is crucial to study the multiple relationships between miRNA–gene target-genes and their roles in various diseases. 

MAGIA^2^ is an updated extension and evolution of MAGIA [27], a web tool for the integrated analysis of miRNAs and gene expression data aimed at the identification of GRNs. MAGIA^2^ reports validated and predicted miRNA–gene interactions from several miRNA databases supporting multiple organisms (human, mouse, rat, and drosophila) and using a large list of prediction algorithms. Although MAGIA^2^ is the most advanced tool currently available for integrative miRNA–gene analyses, it is not able to integrate the information on validated protein–protein or gene–gene interactions, such as those reported by GeneMANIA, or those related to diseases and drugs as those implemented in MultimiR. MAGIA^2^ can estimate some of the relationships between transcription factor (TF)-gene interactions and miRNA–gene interactions, but the predicted regulatory circuits are limited to gene-TF interactions. Conversely, SpidermiR performs an integrative analysis on more comprehensive interaction types, and it provides all the features offered by the other competing tools, also including extracellular and circulating miRNAs and validated gene–gene interactions.

Compared to MAGIA^2^, SpidermiR, has other advantages: (1) to identify regulatory circuits, users do not need to locally download the gene/miRNA expression data for each analysis; (2) since it is open software available in Bioconductor, users can integrate other analyses that are not already implemented in the tool; and (3) users can integrate miRNA–gene interaction into different network data, such as co-expression, physical interactions, genetic interactions, shared protein domains, co-localization and pathways, and are able to select miRNA–GRNs communities.

In addition, compared to online web portal, packages in the statistical software R provide more user-controlled features with no limitations on the number of miRNA/genes to be used.

The particular features (and sub features) of GeneMANIA, mirnaTap, multiMiR, MAGIA^2^, and SpidermiR tools are summarized in Table 1. 

### 2.3. Case Study No. 1. Prostate Cancer: Role of miRNAs in Shared Protein Domains

In this case study, we illustrate an application of SpidermiR aimed at identifying miRNAs that control shared protein domains in aggressive prostate cancer (PC). Combining gene expression data on aggressive PC samples from TCGA and shared protein domains from GeneMANIA, SpidermiR enabled us to detect a community of shared protein domains enriched by DEGs with direct gene-gene interactions, regulated by specific miRNAs. These miRNAs regulators could be interesting as therapeutic tools in aggressive PC.

Domains are generally considered as the conserved structural and functional units of proteins, and previous studies have shown that about 80% of protein pairs sharing the same domain also share the same function and have an a high degree of sequence similarity [28,29]. In the literature, there are several examples of proteins with similar domains, which are involved in cancer development: for instance, the protein family of Src, containing among other proteins Fyn and Yes, possesses all SH domains that are mutated in breast cancer, colon cancer, head and neck carcinoma, and non-small cell lung cancer [30].

We started with a network of shared protein domain interactions, consisting of 16,502 nodes (proteins) and 1,041,003 edges (interactions)—using SpidermiRquery, SpidermiRdownload, and SpidermiRprepare—and we enriched this network with miRNA-gene interactions, including only miRNAs already found to be deregulated in PC according to the literature, using SpidermiRanalyze_mirna_gene_complnet. This second network consisted of 13,338 nodes, (74 miRNAs and 13,264 genes) and 100,016 edges.

We identified 421 DEGs from TCGA data between aggressive PC (Gleason Sore ≥ 7; *n* = 452) versus normal samples (*n* = 52), and we integrated DEGs into the second network, thus obtaining a third network of only 80 nodes (13 miRNAs and 67 genes) and 86 edges, containing DEGs which interact directly, using SpidermiRanalyze_DEnetworkTCGA [25,26].

**Figure 2:**
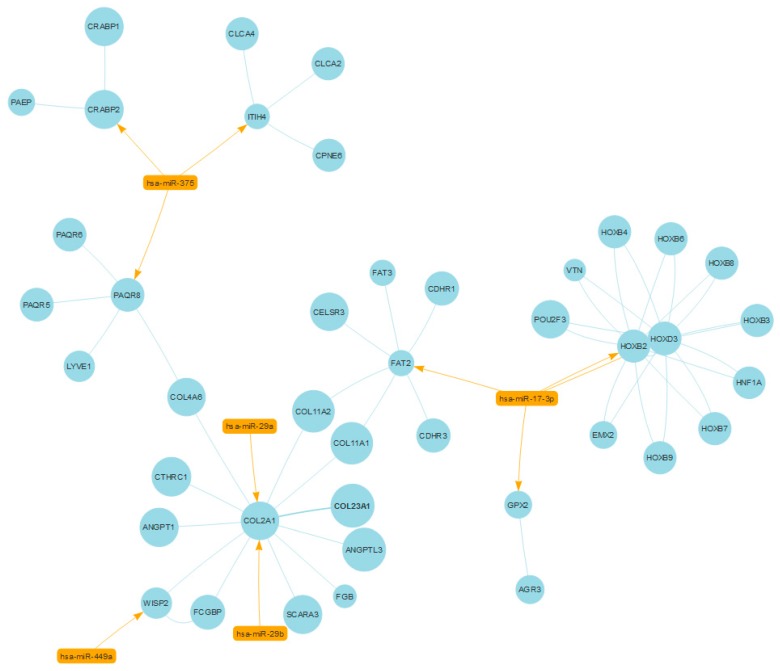
A community of shared protein domains enriched by 42 interacting genes/proteins (blue color) differentially expressed in aggressive prostate cancer (PC), and regulated by five miRNAs (orange color) (using SpidermiRvisualize_mirnanet [25,26]). Three of these genes/proteins were found with a high degree of centrality and thus they are hubs of the network.

This network was processed to find communities based on density metrics and we then focused on the community with the highest number of elements, resulting in a subnetwork of 47 nodes (5 miRNAs and 42 genes) and 59 edges (fourth network), using SpidermiRanalyze_Community_detection and SpidermiRanalyze_Community_detection_net. Three nodes were miRNAs with direct interactions with three genes with a high centrality (hubs), which are directly connected to all the other genes in the community. The modulation of these three miRNAs could help regulate the community, thus pointing to these miRNAs as potential therapeutic tools in aggressive PC. A graphical visualization of these communities is presented in Figure 2.

miR-17-3p has already been found to play a role in PC growth and invasion [31], and has been proposed as a biomarker secreted in the urine of PC patients [5]. miR-29a and b have been proposed as tumor suppressors in PC [32,33,34]. 

The *HOX* gene family includes various transcription factors that help to determine cell and tissue identity in the early development of PC [35], and increase greatly during the development of PC [36]. 

*FAT2* belongs to those Cadherin superfamily members that are homologous to Drosophila Fat, which functions as a regulator of Drosophila wing development [37]. *FAT2* has been described as a regulator of invasiveness in breast cancer [38]. The literature has not yet clarified the role of *FAT* genes in PC, nor in other types of human cancers, where they could act as a controller of the invasion ability of the tumor cells by regulating the FAT Hippo signaling cascades [39].

Prostatic calcification is commonly observed and has been associated with PC [40]. Studies on tumor endothelial cells isolated from mice that spontaneously developed PC revealed that the calcification process in prostate vessels is sustained by the chondrogenic differentiation of endothelial cells. This population of cells, which is differentiated from chondrocytes, expresses increased levels of cartilage-specific markers, such as the type II collagen (*COL2A1*) gene, thus encoding the extracellular matrix protein type II collagen [41]. This gene was found in our analysis as one of the potential hubs regulated by miR-29a/b.

In addition several members of the *HOX* family (e.g., *HOXB6*, *B7*) that belong to this subnetwork, are involved in PC onset [35] or development [36].

### 2.4. Case Study No. 2. Breast Cancer: The Role of miRNAs in Regulating High Degree Centrality Proteins in Physical Interactions

In this case study, we illustrate an application of SpidermiR aimed at identifying a network of key miRNAs involved in breast cancer (BC), which directly interact with proteins in physical interaction (PI). PIs are referred to as protein–protein interactions, and are crucial for assessing the structural and functional architecture of the cell in terms of how direct associations between molecules constitute protein complexes, signal transduction pathways and other cellular machinery [42]. There is a positive correlation between protein connectivity, described by PI parameter, and regulatory miRNAs, as demonstrated by Liang et al. [43]. Moreover, to assess protein connectivity, degree centrality parameter is a key measure. Proteins with a high degree centrality are usually more essential for the survival and growth of the organism than proteins with low degree centrality values [9,43]. However, to our knowledge, there is no comprehensive study on the relationship between proteins with high degree centrality and their regulating miRNAs with high degree centrality in this case study, we assessed the hypothesis that miRNAs with a high degree centrality may preferably regulate proteins with high degree centrality.

We downloaded PI data in *Homo sapiens* from GeneMANIA, and we obtained a first network with 15,407 nodes (all proteins) and 189,030 edges, using SpidermiRquery, SpidermiRdownload, and SpidermiRprepare. We integrated miRNAs already found as deregulated in BC in the literature within this network, thus obtaining a second network of miRNA-protein PI including 830 nodes (62 miRNAs and 768 proteins) and 1001 edges, by using SpidermiRanalyze_mirna_network [25,26].

**Figure 3:**
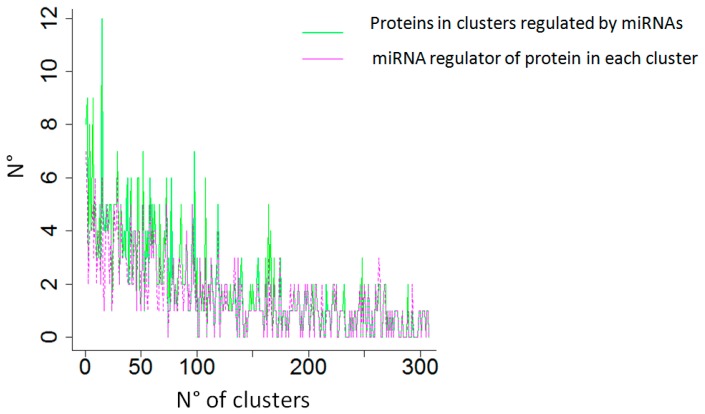
Pink color: number of miRNAs regulators of proteins with the highest degree of centrality in clusters. Green color: number of proteins in clusters regulated by miRNAs with the highest degree of centrality. Clusters are ordered according to their degree centrality (Cluster 1 = Highest degree centrality).

Next, the degree centrality of the proteins in the first network and of miRNAs in the second network were computed by using the function SpidermiRanalyze_degree_centrality. Proteins were clustered according to their degree centrality (50 proteins/cluster), identifying more than 300 clusters of proteins, and we then assessed the relationships between miRNAs with higher centrality and their target proteins with higher centrality. Statistical results showed that proteins with higher centrality are effectively targets of miRNAs with higher centrality (Pearson correlation coefficient = 0.7) (see Figure 3). We then focused on the cluster of proteins with the highest degree centrality (cluster No. 1): we found eight proteins were interacting with seven miRNAs with a high degree of centrality (see Table 2).

**Figure 4:**
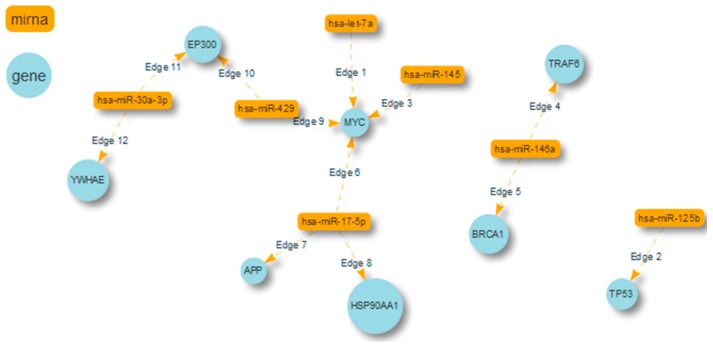
Representation of the eight proteins interacting with seven miRNAs with SpidermiRvisualize.

Figure 4 shows this network, describing the key miRNAs involved in BC and directly interacting with proteins in PI (only the direct interactions among BIs of the network are shown). For some of the identified miRNAs, the regulation of genes with a high degree of centrality has already been demonstrated. For instance, let-7a [44] and miR-145 [45] are known regulators of the high degree of centrality protein MYC, and miR-125b is the controller of the high degree of centrality protein p53 [46]. miR-17-5p, the miRNA with the highest degree centrality in the network, has V-Myc Avian Myelocytomatosis Viral Oncogene Homolog (MYC), Amyloid β Precursor Protein (APP), and HSP90AA1 as target mRNAs, which also present a high degree of centrality.

## 3. Materials and Methods

The SpidermiR package consists of functions that can be grouped into three main levels: (i) data; (ii) analysis; and (iii) visualization. In the following sections, we briefly describe the pipelines that can be used in case studies through these functions. 

### 3.1. Data

The SpidermiR data function includes three main function categories: (i) query; (ii) download; and (iii) annotation harmonization.

(i) “Query” enables users to query: (1) recent and archived data and to identify the elements to download; and (2) species and network type via GeneMANIA database.
(ii) “Download” enables users to download: (1) gene–gene networks as previously queried; (2) miRNA validated data targets using miRTar and miRWalk databases [10,11]; (3) miRNA predicted data targets using DIANA, Miranda, PicTar, and TargetScan databases [12,13,14,15]; (4) extracellular/circulating miRNAs using the miRandola database [16]; (5) the associations among miRNAs, genes, and diseases, using the miR2Disease database [17], and among miRNAs, genes and drugs, using the Pharmaco-miR database [18].
(iii) “Harmonization” enables users to process the data for downstream analyses and it prepares a matrix of gene networks by mapping Ensembl Gene ID to gene symbols. Gene symbols are needed to integrate miRNAdata.

### 3.2. Analyses

The analysis functions are designed to process network data through standard and novel computational methods. Once the network data have been prepared with Gene symbols ID, the downstream analyses can be divided into: (i) enrichment; (ii) interaction selection; and (iii) community detection.

(i) “Enrichment” enables users to: (1) enrich the networks with some further biological information. For example, for each network users can integrate miRNA databases (validated or predicted) in order to find miRNA–gene target interactions in the downloaded gene network; (2) retrieve the information on miRNA–gene and gene-pharmaco from the Pharmaco-miR database; (3) retrieve the extracellular/circulating miRNA database in order to find miRNA–gene target interactions in the downloaded gene network; (4) enrich a chosen network with DEGs. Users can simply choose the type of tumor, platform, and the ID samples from the TCGA portal and then obtain the directed interactions of DEG among them [23,24].
  In the enrichment step, SpidermiR combines interactions found in all validated databases, and it combines only interactions commonly found in at least two predicted databases.
(ii) “Interaction Selection”. In this step, users can play with the obtained network. For example, user can: (1) find sub-networks including all direct interactions involving at least one of the biomarkers of interest (BIs)—this is carried out on the basis of a set of BIs, genes, miRNA, or both; (2) search for sub-networks including all direct interactions involving only BIs; (3) can search for sub-networks including all direct and indirect interactions involving at least one of the BIs; (4) find the number of direct neighbors of a BI and select those BIs with a number of direct neighbours higher than a given cut-off value.
(iii) “Community detection”. In this step, users can analyze the network to detect communities using algorithms developed in the study by Csardi et al. [47], and characterize them in terms of the number of community elements (both genes and miRNAs). On the basis of a community to which some BIs belong, the community can be characterized as a network of elements (both genes and miRNAs), and users can find out whether or not a set of BIs is included within such a community.

### 3.3. Visualization

The visualization functions enable users to display the results of the analyses through a graphical representation of networks (i.e., with vertices, nodes and edges) and plots (for other results).

For example, users can see a 3D representation of the network [48] in different colors for miRNAs, genes, and drugs, and manage the network directly by moving/shifting the nodes and the edges, according to the analysis and visualization needs. Users can highlight specific BIs within a network with a different color.

Some metrics are computed and plotted for the network, such as the number of direct neighbors of BI (i.e., the degree centrality), the cumulative frequency distribution of degree centrality of communities, and the adjacency matrix of the community, representing the degree of connections among the nodes. A summary of the networks is also provided showing the number of edges, nodes, and miRNAs.

## 4. Conclusions

The huge amount of data on validated biological networks currently made available in public repositories offers an excellent opportunity to interpret molecular mechanisms and molecular dynamics, as well as to promote discoveries of novel diagnoses, prognoses, treatments, and monitoring protocols to improve clinical outcomes for patients.

However, the tools available to exploit these data are not exhaustive, and do not offer the user a workflow to download, integrate, and/or analyze the data with miRNAs in an environment that can also provide access to other statistical analysis methods, such as those provided by the increasingly popular Bioconductor repository.

In this paper we have presented SpidermiR, a software tool freely available on the Bioconductor platform, which provides functions to query, download, and process biological network data.

With the various functions, users can incorporate additional biological information retrieved from public databases in the selected network, such as miRNA data (validated, predicted, and extracellular circulating miRNA), disease annotations, drug associations, and TCGA data.

In addition, using interactive and community detection users can apply standard and novel methods for the analysis of miRNA-gene-gene interactions, and this is especially useful in the field of genomics and epigenomics research, also in identifying diagnostic and prognostic biomarkers for specific tumors and simulating candidate miRNAs as therapeutic agents. The visualization functions enable users to display the results generated by the analyses through a graphical representation of networks.

These functions provide an easy, time-saving computational instrument for complex investigations, without having to navigate through different web and data portals and integrating different computational platforms.

Our two case studies highlight the typical uses of our package, such as protein-protein interactions (shared_protein_domains and physical_interactions), gene–miRNA interactions, gene-gene–miRNA, miRNA disease associations, TCGA data, community detection, and degree centrality. Our studies also demonstrate how the package can generate candidate biomarkers, and network communities and their miRNA regulators, which could subsequently be tested experimentally in the laboratory.

## Figures and Tables

**Table 1:** Each column represents a software tool, and each row represents a feature. The checked cells indicate features that are implemented in the tool. Available platform abbreviations are defined as: R (only R script), B (integrated in Bioconductor package), and W (available as a web portal). * only gene-transcriptional factor, ** co-expression, genetic interaction, co-localization, and pathway, *** physical interactions, shared protein domain.

Features	Sub-Features	SpidermiR	GeneMANIA	miRNATap	multiMir	Magia^2^
Availability	Platform	B	W	B	R	W
	Functions for data query/download/annotation harmonization	●		●	●	●
	Functions for GRNs enrichment	●				●
	Functions for interaction selection	●				●
	Functions for community detection	●				
	Functions for miRNA–GRNs graphics	●				●
	Functions for computation of miRNA–GRNs metrics	●				●
	Expression level	●				●
Interaction Type	Predicted miRNA–gene	●		●	●	●
	Validated miRNA–gene	●			●	●
	Disease–miRNA	●			●	
	miRNA–gene–drug	●			●	
	Extracellular/circulating miRNA	●				
	Predicted gene–gene	●	●			● *
	Validated gene–gene	● **	● **			
	Validated protein–protein	● ***	● ***			
	Validated gene–gene–miRNA	●				
	Validated protein–protein–miRNA	●				
	Predicted gene–gene–miRNA	●				● *

**Table 2:** The 10 top miRNAs with the highest degree of centrality (d.c.) and their target proteins with the highest degree of centrality.

miRNA	Target Protein
miR-17-5p (d.c.296)	APP (d.c. 2008) HSP90AA1 (d.c. 773) MYC (d.c. 570)
miR-125b (d.c.55)	TP53 (d.c. 630)
miR-146a (d.c.38)	TRAF6 (d.c. 517) BRCA1 (d.c. 416)
miR-30a-3p (d.c.28)	EP300 YWHAE (d.c. 390)
let-7a (d.c. 26)	MYC (d.c. 570)
miR-429 (d.c.26)	MYC EP300 (d.c. 492)
miR-145 (d.c.23)	MYC

## Abbreviations

GRNs	Gene Regulatory Networks
miRNAs	MicroRNAs
TCGA	The Cancer Genome Atlas
PC	Prostate cancer
BC	Breast cancer
PI	Physical interaction
BI	Biomarker of interest

## References

[1] Warde-Farley D., Donaldson S.L., Comes O., Zuberi K., Badrawi R., Chao P., Franz M., Grouios C., Kazi F., Lopes C.T. (2010). The GeneMANIA prediction server: Biological network integration for gene prioritization and predicting gene function. Nucleic Acids Res..

[2] Jacobsen A., Silber J., Harinath G., Huse J.T., Schultz N., Sander C. (2013). Analysis of microRNA-target interactions across diverse cancer types. Nat. Struct. Mol. Biol..

[3] Flynt A.S., Lai E.C. (2008). Biological principles of microRNA-mediated regulation: Shared themes amid diversity. Nat. Rev. Genet..

[4] Bertoli G., Cava C., Castiglioni I. (2015). MicroRNAs: New Biomarkers for Diagnosis, Prognosis, Therapy Prediction and Therapeutic Tools for Breast Cancer. Theranostics.

[5] Bertoli G., Cava C., Castiglioni I. (2016). MicroRNAs as Biomarkers for Diagnosis, Prognosis and Theranostics in Prostate Cancer. Int. J. Mol. Sci..

[6] Yuan X., Liu C., Yang P., He S., Liao Q., Kang S., Zhao Y. (2009). Clustered microRNAs′ coordination in regulating protein–protein interaction network. BMC Syst. Biol..

[7] Lim L.P., Lau N.C., Garrett-Engele P., Grimson A., Schelter J.M., Castle J., Bartel D.P., Linsley P.S., Johnson J.M. (2005). Microarray analysis shows that some microRNAs downregulate large numbers of target mRNAs. Nature.

[8] Lancichinetti A., Kivelä M., Saramäki J., Fortunato S. (2010). Characterizing the community structure of complex networks. PLoS ONE.

[9] Jeong H., Mason S.P., Barabási A.L., Oltvai Z.N. (2001). Lethality and centrality in protein networks. Nature.

[10] Hsu J.B., Chiu C.M., Hsu S.D., Huang W.Y., Chien C.H., Lee T.Y., Huang H.D. (2011). miRTar: An integrated system for identifying miRNA-target interactions in human. BMC Bioinform..

[11] Dweep H., Sticht C., Pandey P., Gretz N. (2011). miRWalk—Database: Prediction of possible miRNA binding sites by “walking” the genes of three genomes. J. Biomed. Inform..

[12] Maragkakis M., Vergoulis T., Alexiou P., Reczko M., Plomaritou K., Gousis M., Kourtis K., Koziris N., Dalamagas T., Hatzigeorgiou A.G. (2011). DIANA-microT Web server upgrade supports Fly and Worm miRNA target prediction and bibliographic miRNA to disease association. Nucleic Acids Res..

[13] Enright A.J., John B., Gaul U., Tuschl T., Sander C., Marks D.S. (2003). MicroRNA targets in Drosophila. Genome Biol..

[14] Krek A., Grün D., Poy M.N., Wolf R., Rosenberg L., Epstein E.J., MacMenamin P., da Piedade I., Gunsalus K.C., Stoffel M. (2005). Combinatorial microRNA target predictions. Nat Genet..

[15] Bartel D.P. (2009). MicroRNAs: Target recognition and regulatory functions. Cell.

[16] Russo F., Di Bella S., Nigita G., Macca V., Laganà A., Giugno R., Pulvirenti A., Ferro A. (2012). miRandola: Extracellular circulating microRNAs database. PLoS ONE.

[17] Jiang Q., Wang Y., Hao Y., Juan L., Teng M., Zhang X., Li M., Wang G., Liu Y. (2009). miR2Disease: A manually curated database for microRNA deregulation in humandisease. Nucleic Acids Res..

[18] Rukov J.L., Wilentzik R., Jaffe I., Vinther J., Shomron N. (2014). Pharmaco-miR: Linking microRNAs and drug effects. Brief. Bioinform..

[19] Pajak M., Simpson T.I. miRNAtap:miRNAtap:microRNA Targets—Aggregated Predictions. https://bioconductor.org/packages/release/bioc/html/miRNAtap.html.

[20] Ru Y., Kechris K.J., Tabakoff B., Hoffman P., Radcliffe R.A., Bowler R., Mahaffey S., Rossi S., Calin G.A., Bemis L. (2014). The multiMiR R package and database: Integration of microRNA–target interactions along with their disease and drug associations. Nucleic Acids Res..

[21] Bisognin A., Sales G., Coppe A., Bortoluzzi S., Romualdi C. (2012). MAGIA²: From miRNA and genes expression data integrative analysis to microRNA-transcription factor mixed regulatory circuits (2012 update). Nucleic Acids Res..

[22] Weinstein J.N., Collisson E.A., Mills G.B., Shaw K.R., Ozenberger B.A., Ellrott K., Shmulevich I., Sander C., Stuart J.M., Cancer Genome Atlas Research Network (2013). The Cancer Genome Atlas Pan-Cancer analysis project. Nat. Genet..

[23] Colaprico A., Silva T.C., Olsen C., Garofano L., Cava C., Garolini D., Sabedot T.S., Malta T.M., Pagnotta S.M., Castiglioni I. (2016). TCGAbiolinks: An R/Bioconductor package for integrative analysis of TCGA data. Nucleic Acids Res..

[24] Silva T.C., Colaprico A., Olsen C., D’Angelo F., Bontempi G., Ceccarelli M., Noushmehr H. (2016). TCGA Workflow: Analyze cancer genomics and epigenomics data using Bioconductor packages [version 1; referees: 1 approved, 1 approved with reservations]. F1000Research.

[25] Cava C., Colaprico A., Graudenzi A., Bertoli G., Silva T.C., Olsen C., Noushmehr H., Bontempi G., Mauri G., Castiglioni I. SpidermiR: Application Examples. https://www.bioconductor.org/packages/release/bioc/vignettes/SpidermiR/inst/doc/SpidermiRcasestudy.pdf.

[26] Cava C., Colaprico A., Graudenzi A., Bertoli G., Silva T.C., Olsen C., Noushmehr H., Bontempi G., Mauri G., Castiglioni I. Working with SpidermiR package. https://www.bioconductor.org/packages/release/bioc/vignettes/SpidermiR/inst/doc/SpidermiR.html.

[27] Sales G., Coppe A., Bisognin A., Biasiolo M., Bortoluzzi S., Romualdi C. (2010). MAGIA, A web-based tool for miRNA and Genes Integrated Analysis. Nucleic Acids Res..

[28] Cohen-Gihon I., Nussinov R., Sharan R. (2007). Comprehensive analysis of co-occurring domain sets in yeast proteins. BMC Genom..

[29] Hegyi H., Gerstein M. (2001). Annotation transfer for genomics: Measuring functional divergence in multi-domain proteins. Genome Res..

[30] Sen B., Johnson F.M. (2011). Regulation of SRC family kinases in human cancers. J. Signal Transduct..

[31] Yang X., Du W.W., Li H., Liu F., Khorshidi A., Rutnam Z.J., Yang B.B. (2013). Both mature miR-17-5p and passenger strand miR-17-3p target TIMP3 and induce prostate tumor growth and invasion. Nucleic Acids Res..

[32] Nishikawa R., Goto Y., Kojima S., Enokida H., Chiyomaru T., Kinoshita T., Sakamoto S., Fuse M., Nakagawa M., Naya Y. (2014). Tumor-suppressive microRNA-29s inhibit cancer cell migration and invasion via targeting LAMC1 in prostate cancer. Int. J. Oncol..

[33] Li J., Wan X., Qiang W., Li T., Huang W., Huang S., Wu D., Li Y. (2015). MiR-29a suppresses prostate cell proliferation and induces apoptosis via KDM5B protein regulation. Int. J. Clin. Exp. Med..

[34] Ru P., Steele R., Newhall P., Phillips N.J., Toth K., Ray R.B. (2012). miRNA-29b suppresses prostate cancer metastasis by regulating epithelial-mesenchymal transition signaling. Mol. Cancer Ther..

[35] Javed S., Langley S.E. (2014). Importance of *HOX* genes in normal prostate gland formation, prostate cancer development and its early detection. BJU Int..

[36] Morgan R., Boxall A., Harrington K.J., Simpson G.R., Michael A., Pandha H.S. (2014). Targeting HOX transcription factors in prostate cancer. BMC Urol..

[37] Katoh Y., Katoh M. (2006). Comparative integromics on FAT1, FAT2, FAT3 and FAT4. Int. J. Mol. Med..

[38] Dang T.T., Westcott J.M., Maine E.A., Kanchwala M., Xing C., Pearson G.W. (2016). ΔNp63α induces the expression of FAT2 and Slug to promote tumor invasion. Oncotarget.

[39] Katoh M. (2012). Function and cancer genomics of FAT family genes (review). Int. J. Oncol..

[40] Smolski M., Turo R., Whiteside S., Bromage S., Collins G.N. (2015). Prevalence of prostatic calcification subtypes and association with prostate cancer. Urology.

[41] Dudley A.C., Khan Z.A., Shih S.C., Kang S.Y., Zwaans B.M., Bischoff J., Klagsbrun M. (2008). Calcification of multipotent prostate tumor endothelium. Cancer Cell.

[42] Beyer A., Bandyopadhyay S., Ideker T. (2007). Integrating physical and genetic maps: From genomes to interaction networks. Nat. Rev. Genet..

[43] Liang H., Li W.H. (2007). MicroRNA regulation of human protein protein interaction network. RNA.

[44] Lyu S., Yu Q., Ying G., Wang S., Wang Y., Zhang J., Niu Y. (2014). Androgen receptor decreases CMYC and KRAS expression by upregulating let-7a expression in ER−, PR−, AR+ breast cancer. Int. J. Oncol..

[45] Kim S.J., Oh J.S., Shin J.Y., Lee K.D., Sung K.W., Nam S.J., Chun K.H. (2011). Development of microRNA-145 for therapeutic application in breast cancer. J. Control. Release.

[46] Maqbool R., Rashid R., Ismail R., Niaz S., Chowdri N.A., Hussain M.U. (2015). The carboxy-terminal domain of connexin 43 (CT-Cx43) modulates the expression of p53 by altering miR-125b expression in low-grade human breast cancers. Cell. Oncol..

[47] Csardi G., Nepusz T. (2006). The igraph software package for complex network research. Int. J. Complex Syst..

[48] Gandrud C., Allaire J.J., Russell K., Lewis B.W., Kuo K., Sese C., Ellis P., Owen J., Rogers J. NetworkD3: D3 JavaScript Network Graphs from R 2015. https://CRAN.R-project.org/package=networkD3.

